# Quantitative Assessment of Brix in Grafted Melon Cultivars: A Machine Learning and Regression-Based Approach

**DOI:** 10.3390/foods13233858

**Published:** 2024-11-29

**Authors:** Uğur Ercan, Ilker Sonmez, Aylin Kabaş, Onder Kabas, Buşra Calık Zyambo, Muharrem Gölükcü, Gigel Paraschiv

**Affiliations:** 1 Department of Informatics, Akdeniz University, 07070 Antalya, Türkiye; ugurercan@akdeniz.edu.tr; 2 Department of Soil Science and Plant Nutrition, Faculty of Agriculture, University of Akdeniz, 07070 Antalya, Türkiye; ilkersonmez@akdeniz.edu.tr (I.S.); bcalik@akdeniz.edu.tr (B.C.Z.); 3 Department of Organic Farming, Manavgat Vocational School, Akdeniz University, 07070 Antalya, Türkiye; 4 Department of Machine, Technical Science Vocational School, Akdeniz University, 07070 Antalya, Türkiye; okabas@akdeniz.edu.tr; 5 Bati Akdeniz Agricultural Research Institute, 07100 Antalya, Türkiye; muharrem.golukcu@tarimorman.gov.tr; 6 Department of Biotechnical Engineering, Faculty of Biotechnical Engineering, National University of Science and Technology Politehnica Bucharest, 060042 Bucharest, Romania

**Keywords:** SVR, grafting, melon, Brix

## Abstract

The article demonstrates the Brix content of melon fruits grafted with different varieties of rootstock using Support Vector Regression (SVR) and Multiple Linear Regression (MLR) model approaches. The analysis yielded primary fruit biochemical measurements on the following rootstocks, Sphinx, Albatros, and Dinero: nitrogen, phosphorus, potassium, calcium, and magnesium. Established models were evaluated with Mean Absolute Error (MAE), Mean Absolute Percentage Error (MAPE), Mean Square Error (MSE), Root Mean Square Error (RMSE), and Coefficient of Determination (R^2^) metrics. In the test section, the results of the MLR model were calculated as MAE: 0.0728, MAPE: 0.0117, MSE: 0.0088, RMSE: 0.0936, and R^2^: 0.9472, while the results of the SVR model were calculated as MAE: 0.0334, MAPE: 0.0054, MSE: 0.0016, RMSE: 0.0398, and R^2^: 0.9904. Despite both models performing well, the SVR model showed superior accuracy, outperforming MLR by 54% to 82% in terms of predictions. The relationships between Brix levels and various nutrients, such as sucrose, glucose, and fructose, were found to be strong, while titratable acidity had a minimal effect. SVR was found to be a more reliable, non-destructive method for melon quality assessment. These findings revealed the relationship between Brix and sugar levels on melon quality. The study highlights the potential of these machine learning models in optimizing the rootstock effect and managing melon cultivation to improve fruit quality.

## 1. Introduction

Melon (*Cucumis melo* L.), which belongs to *Cucurbitaceae* family, is a eudicot diploid plant species (2n = 2x = 24) and it is originally from Asia [1]. It is one of the most widely cultivated crops in the world and in Türkiye, and grows best in warm temperate climates. According to FAO 2023, global melon production reached 28,467,920 tons on an area of 1,068,238 hectares, while Türkiye produces 1,724,856 tons on 76,129 hectares [2]. Most cucurbit fruits are an important source of vitamin C, provitamin A, toxic folic acid, phenolic phytochemicals, dietary fibers, and minerals. Cucurbitacin, lithium, and zinc in these ingredients help prevent cancer, fight depression, protect from ulcers, and regulate the immune system [3,4,5]. Melon is also rich in bioactive compounds that are important for human health, and it contains carotenoid antioxidants such as beta-carotene (provitamin A) as well as ascorbic acid, carotene, folic acid, and potassium. It also has anti-carcinogenic effects and is reported to prevent cardiovascular disease, cataracts, and night blindness [6]. Melon fruits have carotenoids and chlorophylls as major pigments [7].

Grafting is a widely used cultural practice in tomato, eggplant, pepper, watermelon, melon, and cucumber production to control soil-borne diseases and improve abiotic stress tolerance in many parts of Asia and Europe [8,9,10]. It is defined as the process of combining parts taken from two plants into a single plant by combining them with certain scientific techniques [9]. Grafting influences various fruit quality attributes, including morphological characteristics (such as fruit shape, color, and size) and internal quality traits, such as chemical composition and sensory qualities of the fruits [11]. For melon, various grafting methods have been employed, but commercial seedling nurseries have recently adopted the tube grafting technique as the preferred method [12]. Interspecific hybrids of *Cucurbita* (*Cucurbita maxima* × *Cucurbita moschata*) are commonly used for grafting cucumber, melon, and watermelon [13]. Grafting plays a pivotal role in the regulation of primary and secondary metabolites of fruit. Grafting provides noticeable increases in fruit yield in many fruit vegetables. It was determined that fresh fruit weight increased in grafted melons compared to non-grafted ones [9].

Brix (total soluble solid) content constitutes a critical quality metric in melons, significantly influencing consumer preferences and the overall marketability of the fruit. It acts as an indicator of the concentration of soluble solids, predominantly comprising sugars, and exhibits a strong correlation with sweetness and flavor intensity. A study conducted by Lester et al. [14] and published in HortScience indicated that elevated Brix levels in melons correlate with enhanced flavor and heightened consumer satisfaction. Research conducted by Fallik et al. [15] in the Journal of the American Society for Horticultural Science demonstrated that Brix content can exhibit considerable variability among distinct melon cultivars and is subject to modulation by factors including cultivation conditions, timing of harvest, and post-harvest management practices. Moreover, Paris et al. [16], in a thorough review published in *Plant Breeding Reviews*, underscored the significance of Brix as a pivotal characteristic in melon breeding initiatives, accentuating its role in the creation of novel varieties characterized by superior sweetness and flavor attributes. Consequently, comprehending and optimizing Brix content is essential for melon producers and breeders in order to fulfill market requirements and enhance fruit quality.

The relationship between melon rootstocks and Brix content is of great importance since rootstocks can significantly alter fruit quality measurements, including sugar concentration. Colla et al. [17] demonstrated that the practice of grafting melons onto specific rootstocks could enhance the Brix content of fruit in comparison to non-grafted specimens, particularly under saline environmental conditions. This phenomenon was attributed to the improved efficacy of water and nutrient absorption. Nevertheless, the influence of rootstocks on Brix content may exhibit variability. Fallik and Ilic [18] noted that while certain rootstocks were effective in augmenting Brix levels, others demonstrated negligible effects or even a reduction in sugar concentration, underscoring the critical nature of rootstock selection. Rouphael et al. [19] indicated that the impact of rootstocks on Brix content could be influenced by various environmental and cultivation practices. Notably, Zhao et al. [20] illustrated that the effects of rootstocks on Brix may be contingent upon the cultivar, with particular melon varieties exhibiting more pronounced reactions to grafting than their counterparts.

The methodologies employed for Brix determination have undergone significant advancements in recent years, emphasizing non-destructive and expeditious techniques. Although traditional refractometry remains prevalent, innovative technologies have surfaced to improve precision and operational efficiency. Xu et al. [21] demonstrated the effectiveness of hyperspectral imaging combined with machine learning for non-destructive Brix prediction in apples. Similarly, Li et al. [22] investigated the use of terahertz spectroscopy for rapid and accurate measurement of Brix in nectarines. Portable near-infrared (NIR) spectroscopy instruments have become increasingly important for in-field assessment, as demonstrated by Hao et al.’s [23] research on kiwifruit. For more meticulous laboratory analyses, high-performance liquid chromatography (HPLC) continues to be optimized, with Zhao et al. [24] documenting an enhanced methodology for the concurrent determination of sugars and organic acids in various fruits.

Machine learning (ML) and Multiple Linear Regression (MLR) have emerged as indispensable methodologies for elucidating Brix content in melons, attributable to their proficiency in managing intricate, non-linear interconnections among diverse fruit attributes and sugar concentrations. These analytical techniques facilitate the amalgamation of numerous input variables, encompassing spectral data, physical characteristics, and environmental parameters, thereby enabling more precise predictions of Brix levels compared to traditional linear frameworks. In the domain of melon research, the integration of near-infrared spectroscopy (NIRS) data with ML algorithms has been utilized to formulate non-destructive assessment methodologies for evaluating fruit quality [25,26,27].

In this study, the aim was to estimate and comparatively examine the Brix amounts in species obtained by grafting on different melon rootstocks using machine learning and Multiple Linear Regression methods. The objective of the present article is to illustrate the efficiency of both SVR and MLR models for the reliable prediction of Brix content in grafted melons onto several rootstocks. The investigation covers the biochemical parameters, such as nutrient elements and sugars, responsible for the Brix level, with a focus on the development of a non-destructive prediction method for the estimation of Brix. Through comparative model analysis, the study aims to provide practical guidance to optimize melon cultivation and improve its nutritional value, sweetness, and marketability by evaluating the effects of rootstock selection on fruit quality.

## 2. Materials and Methods

### 2.1. Plant Material

**Figure 1:**
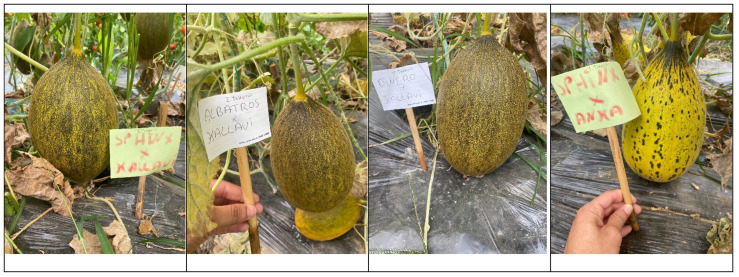

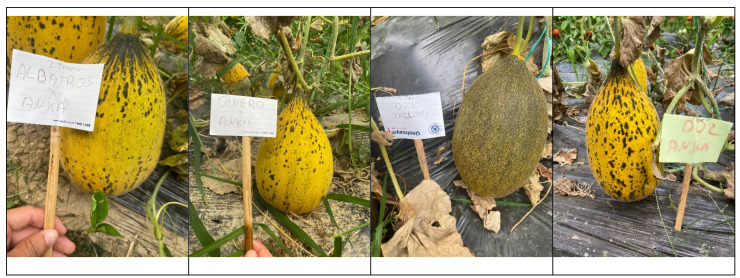
The fruit images of melon varieties from the greenhouse experiment.

This study was conducted in a greenhouse cultivation area in Antalya, Türkiye. The melon cultivars ‘Kallavi’ and ‘Anka’ (Yüksel Seeds Company, Antalya, Türkiye) were used as scions, while three different rootstocks ‘Sphinx’ (Rijk Zwaan, Company, De Lier, The Netherlands), ‘Albatros’ (Rito-Takii Seeds Compnay, Kyoto, Japan), and ‘Dinero’ (Sygenta Seeds Company, Basel, Switzerland) were used as rootstocks. The non-grafted ‘Kallavi’ and ‘Anka’ plants were used as the control (Figure 1). A randomized complete block design with three replications was used in each experimental unit.

#### 2.1.1. Grafting of Melon Seedlings

The tube grafting method was utilized in the experiment. The rootstock and the cultivar had developed true leaves (2–3 leaf stage), and the rootstock seedling was carefully removed from the growing medium without disturbing the substrate, leaving a stem length of 1.0–1.5 cm. The cotyledon leaves were then cut at an angle. Similarly, the cultivar seedling was cut at an angle above the cotyledon leaves, ensuring that the cut surfaces of both the rootstock and cultivar matched appropriately. Grafted seedlings were incubated in a post-grafting growth chamber under 95% humidity at 25 °C [28].

#### 2.1.2. Plant and Fruit Analysis

During the vegetation period, leaves (8th leaf from the tip of growth) that completed their development during the full flowering period (middle stages of the vegetation period) in melon plants were taken as leaf samples. Fruit samples were taken from melons that reached harvest maturity to represent each plant, and the fruit yield and Brix were determined. Element concentrations of leaves and fruits were evaluated separately. For the determination of element concentration, melon leaves and fruits were rinsed with distilled water after washing with tap water and were dried in an air-forced oven at 65 °C until reaching constant weight [29]. After that, dried leaves and fruits were ground for further analysis. Dried leaf and fruit samples of 0.5 g each were digested with 10 mL HNO_3_/HClO_4_ (4:1) acid mixture on a hot plate.

The samples were then heated until a clear solution was obtained, and the same procedure was performed several times. The samples were filtered and diluted to 100 mL using distilled water. The nitrogen concentration (N) was determined by Kjeldahl digestion according to Bremner [30]; concentrations of potassium (K), calcium (Ca), magnesium (Mg), iron (Fe), zinc (Zn), manganese (Mn), and copper (Cu) in the digests were determined using inductively coupled plasma (Perkin Elmer Optima DV7000-ICP OES) [29]. Phosphorus (P) was measured by a spectrophotometer [31]. Mineral element concentration of fruits and leaves were presented in dry weights (DW) as percentages (%) and mg kg^−1^, respectively. 

The pH value was measured with a digital pH meter (Mettler Toledo, SevenEasy, Shanghai, China), and the titratable acidity (TA) was determined by diluting 10 g of homogenized sample with 25 mL of pure water and titrating it with 0.1 N NaOH (sodium hydroxide) until the pH reached 8.1. The results were calculated as % of anhydrous citric acid. Total dry matter amount was determined by drying fresh samples at 70 °C in an oven (Venticell-404 Standard, MMM group, München, Germany) until reaching constant weight, and total ash content was determined by burning off the organic matter part of fresh samples at 550 °C in an ash oven (Protherm, PLF 110/10, Ankara, Türkiye). Total dry matter and ash content were expressed as % of the edible part of the fresh fruit. In order to determine the total soluble solid content, the edible part of the samples was homogenized with ultra-turrax (IKA T25, Brandenburg, Germany) for 1 min and centrifuged at 5000 rpm at 4 °C (Sigma, 2-16KL, Osterode am Harz, Germany). The supernatant was used to determine the total soluble solid content by using a digital refractometer (A. Krüss Optronic GmbH, DR6000 series, Hamburg, Germany) at 20 °C [32]. Total phenolic matter content was determined by using the Spanos and Wrolstad [33] method. Samples were homogenized with ultra-turrax for 1 min and centrifuged at 5000 rpm at 4 °C. The supernatant part was used to determine phenolic matter content. The phenolic content of these parts were measured at 765 nm by using a UV-Vis spectrophotometer (Shimadzu, UV-1800, Tokyo, Japan). The results for phenolic content were expressed as gallic acid equivalents using a standard calibration curve of this phenolic compound. 

The sugar components of the sample were determined from the juice part of the melon pulp, which was centrifuged at 5000 rpm for 5 min at 4 °C, and the supernatant part was diluted with ultra-pure water at a ratio of 1:3. The sugar composition of the samples was determined by an HPLC system (LC 20 AT model, Shimadzu Co., Tokyo, Japan) with a refractive index detector (RID 10A). Separation of sugar was performed using a CarboSep CHO-820 CA column (Transgenomic, Omaha, NE, USA) (7.8 × 300 mm). The HPLC elution was carried out at 80 °C with an isocratic flow of ultra-pure water at a flow rate of 0.5 mL/min and injection volumes of 20 μL [34], and the total analysis time was 20 min. The calculation of each sugar was based on the external standard method from the peak area by analytical interpolation in a standard calibration curve and was expressed as percentages (%). 

### 2.2. Machine Learning

**Figure 2:**
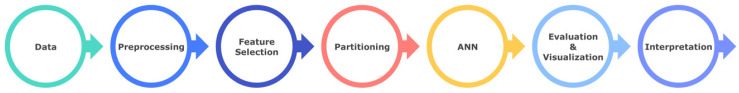
Working flow chart.

Accessing knowledge from data is a process that is iterative and interactive. In these process, data are selected, preprocessed, transformed, modelled, the model results are calculated, visualized, and interpreted, respectively. Also, ML methods are called modeling. The steps shown in Figure 2 were carried out in accordance with the purpose of this study. The flow chart of the work carried out can be seen as follows, in Figure 2.

**Figure 3:**
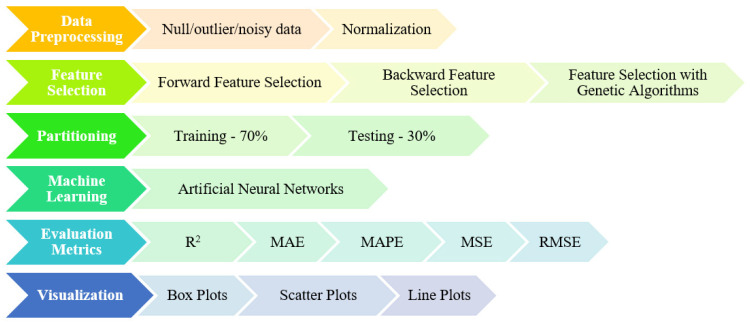
Stages and details of the process from data to knowledge.

Details regarding the operations performed in the main steps are shown in Figure 2, while the process of obtaining knowledge from the data in the study is shown in Figure 3.

The aim of the conducted study is to perform regression using some properties of melon fruit. While performing the regression task, the ANN method, which has been successfully used in regression problems, was used.

#### Support Vector Regression 

Support Vector Machine (SVM), a machine learning technique grounded in statistical learning theory [35], is used to solve classification and regression problems. SVM is a method that classifies given data by finding the optimal hyperplane with the largest margin between classes in a high-dimensional feature space [36]. SVMs are divided into two groups: Support Vector Classification (SVC) if used to solve classification problems, and Support Vector Regression (SVR) if used to solve regression problems. Although they are similar in basic structure, there are some differences between the two methods. In classification problems, the margin of the hyperplane that separates the data sets is maximized to ensure no data points lie within it [37]. In contrast, in regression problems, it is formulated as an optimization problem that minimizes error and seeks the narrowest tube around the surface that encompasses the majority of the training samples [38]. 

The aim of SVR is to find a function *f*(*x*) that predicts the $y_{i}$ value with the most deviation ε (error value) for all training data $x_{i}$ and is also as flat as possible [39]. Support Vector Regression is classified as linear and non-linear. In linear DVR, the *f*(*x*) function is expressed as in Equation (1).

(1) $$f\left(x\right)={w.x}_{i}+b$$

In the equation, *w* represents the weight vector and *b* represents the deviation [40]. In DVR, which was developed to solve non-linear regression problems, it moves the training data in the input space to a higher dimensional space with the help of a non-linear function and applies linear regression in this space. In this case, the representation of the linear function obtained for the best regression is shown in Equation (2).

(2) $$f\left(x,w\right)=\sum_{i=1}^{N}w_{i}\varphi \left(x_{i}\right)+b=w^{T}\varphi \left(x\right)+b$$

In Equation (2), $w\in R^{m}$ denotes the model parameter vector, while the deviation term on the vertical axis is denoted by b∈R. Thus, the linear regression obtained by the dot product between w and *φ*(*x*) in the high-dimensional space corresponds to the non-linear regression in the input space [41]. In non-linear regression models, kernel functions are used to transform the input space into a higher dimensional feature space. Commonly used kernel functions are polynomial, rbf, sigmoid, and linear [42].

In this study, some tuning was made for the SVR model. The parameters that give the best results were C = 3.0, coef0 = 0.2, degree = 2, epsilon = 0.05, and kernel type = poly.

### 2.3. Multiple Linear Regression

Explanation of the dependent variable (in other words, the explained variable) by independent variables (in other words, explanatory variables) by creating a linear model is called regression analysis [43]. Regression models with one dependent variable and one independent variable are called simple regression models. If the number of independent variables in a model is more than one, it is called a Multiple Linear Regression model. The MLR analysis model can be formulated as in Equation (3),

(3) $$Y={\beta_{0}+\beta }_{i}X_{i}+\varepsilon_{i}$$

$\beta_{0}$ is constant, $\beta_{i}$ are parameters, $X_{i}$ are independent variables, Y is a dependent variable, and $\varepsilon_{i}$ is the error term, equal to the difference between the actual values and the predicted values of the dependent variable [44]. For the regression model to be reliable, valid, and usable, it must meet certain conditions. Diagnostic tests are used to check whether these conditions are met and the regression model must pass these tests. Information on the diagnostic tests required for the regression model, the purposes for which they will be used, and the analysis results are given in Table 1 [45,46,47,48].

The evaluation of regression models was conducted using the most commonly employed criteria: MAE, MAPE, MSE, RMSE, and R². Equation representations of the criteria used to evaluate the models and basic information on them are given in Table 2.

## 3. Results and Discussion

**Figure 4:**
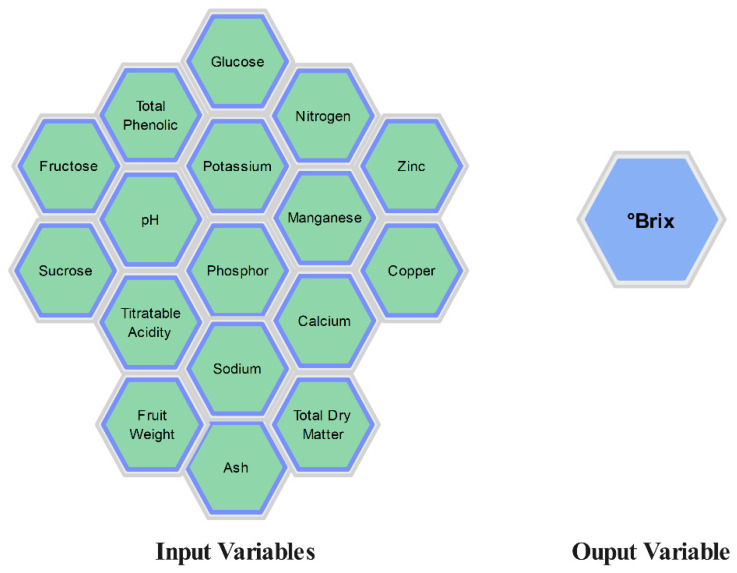
Input and output variables.

The results of the descriptive statistical analysis for the values obtained from the melon fruits used in the study are presented in Table 3. Figure 4 shows the input variables and output variables included in the study.

Table 4 presents the results of the MLR model developed to estimate the Brix content in melon fruit.

Based on the findings of the MLR model developed to predict the Brix content in melon fruit, while nitrogen, phosphorus, potassium, calcium, magnesium, zinc, manganese, copper, pH, dry matter amount, ash, phenol, sucrose, glucose, fructose, and fruit weight are significant, titratable acidity is insignificant. Potassium and fructose are significant at the 5% error level, whereas the other variables are significant at the 1% error level. The Brix model estimated as a result of Multiple Linear Regression analysis can be written as in Equation (4),

(4) $$\begin{array}{ll} \hat{brix}=-2.499 & -0.065N-0.690P+0.112K-2.419Ca+1.970M-0.128Zn\\ & +0.207Mn+0.106Cu+0.655pH+0.212DryMatter\\ & -1.350Ash+0.003TFenol+0.617Sak+1.235Glu\\ & +0.089Fru-0.252FruWei \end{array}$$

A positive correlation was found between °Brix and sugar contents (sucrose, glucose, and fructose) in melon fruits. Since sugars are the most abundant soluble solids in many fruit and vegetable juices, °Brix values determine the estimation of the amount of sugar content in fruits and vegetables [49]. The ripening level of the fruit is related to the °Brix content and °Brix indicates the content of soluble substances in the solution. The components present in fruit include water-soluble components such as glucose, fructose, sucrose, and water-soluble protein (pectin) [50]. Chikh-Rouhou et al. [51] found a negative correlation between °Brix and fruit weight and a positive correlation between total phenolic content, titratable acidity, and pH in melon fruit. Increased °Brix and vitamin C contents were determined in melon with foliar K applications [52,53,54]. Brito et al. [55] found significant responses of phosphorus sources on commercial and total fruit yield, °Brix and soluble solids/total acid ratio in melon fruit, and a negative relationship with Brix. Bhimappa et al. [56] determined a positive correlation between Brix and K and Cu contents and a negative correlation with P content in melon fruit.

An ANOVA test was performed to investigate whether there was a significant difference in Brix amounts according to melon rootstocks. The hypotheses related to this analysis are as follows:

$$H_{0}:\;\mu_{1}=\mu_{2}=\mu_{3}$$

$$H_{A}:\;At\;least\;one\;\mu_{i}\;is\;different$$

In the ANOVA test, the Bartlett test (Bartlett’s test for equal variances) result should be checked first [47]. According to the Bartlett test result, *p* was obtained as 0.717. Since *p* > 0.05, the null hypothesis was accepted and the homogeneity assumption was met. Then, the F and *p* values obtained from the ANOVA test were examined. It was obtained as F: 1.21, *p*: 0.2997. Since *p* > 0.05, the *H_0_* hypothesis that the Brix averages are equal according to melon rootstocks was accepted. In other words, there was no significant difference between the average Brix values according to melon rootstocks. The ANOVA test results are shown in Table 5.

Table 6 shows the results of the Multiple Linear Regression and Support Vector Regression models. For the training data set, the MLR model’s results are MAE: 0.0675, MSE: 0.0076, RMSE: 0.0871, R^2^: 0.9503, and MAPE: 0.0108, and the SVR model’s results are MAE: 0.0299, MSE: 0.012, RMSE: 0.0340, R^2^: 0.9924, and MAPE: 0.0048.

**Figure 5:**
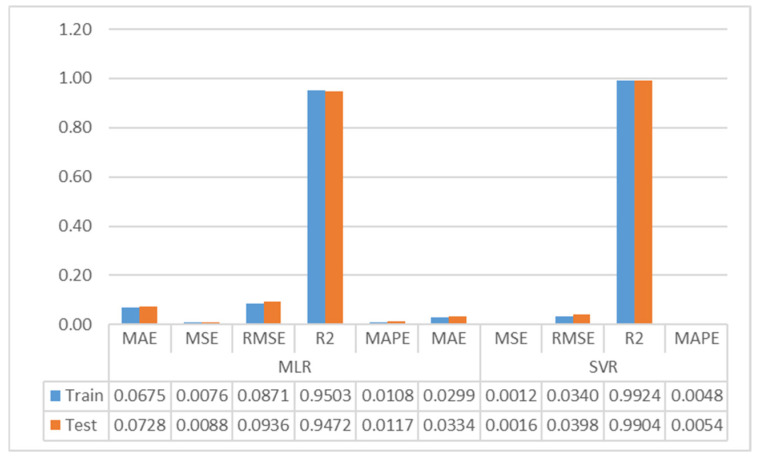
Bar chart of the MLR and SVR models.

The metric results of the SVR and MLR models are shown as a bar chart in Figure 5.

As can be seen in Table 6, Multiple Linear Regression model training and testing partition results are remarkably close each other. The training and testing data set results of the SVR model are similar and consistent. The MLR model had similar results. The results indicate that neither model exhibits an overlearning problem. Based on the test data set results of the MLR model, the Brix quantity in melon fruit is estimated with a Mean Absolute Error of 0.0728, and as a percentage, the Brix quantity can be estimated with an error of 0.0117% (MAPE). The model predicts the Brix content with an RMSE of 0.0936 and an MSE of 0.0088. The variation explained by Multiple Linear Regression model is about 94.7%. Looking at the test data set results of the SVR model, the amount of Brix in melon fruit is estimated by the SVR model with a Mean Absolute Error of 0.0334, while the Brix quantity can be estimated with an error of 0.0054%. While the model estimates the amount of the Brix with an RMSE of 0.0398, the model estimates the amount of the Brix with an MSE of 0.0016. The explained variation by Support Vector Regression is approximately 99%. In the test data set, the SVR model is approximately 54%, 82%, 57%, 54%, and 4% better than the MLR model in MAE, MSE, RMSE, MAPE, and R^2^ metrics, respectively. Both models predict the amount of Brix with high accuracy. Nevertheless, it can be stated that the SVR model provides better predictions than the MLR model. Similar comments can be made within the training partition. 

**Figure 6:**
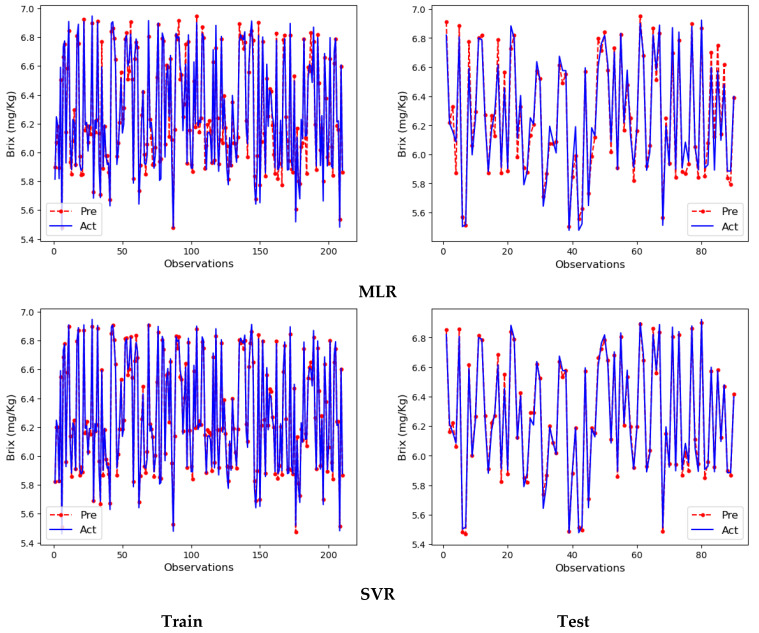
Line plots of the MLR and SVR models.

Line plots created with data obtained from MLR and SVR model results are shown in Figure 6. The plots on the left side were produced with the data obtained from the training partition of the MLR and SVR models, whilst the plots on the right side were produced with the data obtained from the testing partition of the models.

In the line plots of the MLR and SVR models drawn with the training partition results, the actual and prediction lines are almost on top of each other. Since the MLR model made small errors in some observations, the actual and prediction lines showed slight deviations. In contrast, in the SVR model, the actual and prediction lines are almost 100% identical. Therefore, the deviations are much smaller in this line plot. Line plots drawn for the test partition are at the bottom. As can be seen from the overlapping of the actual and prediction lines in the line plots, the models made very good predictions in this section as well. However, the SVR model made better predictions than the MLR model. The deviations made in the estimation of some observations are reflected in the plots. As a result, both training and test partitions predictions are extremely successful, and the results detailed in Table 6 support these visuals.

**Figure 7:**
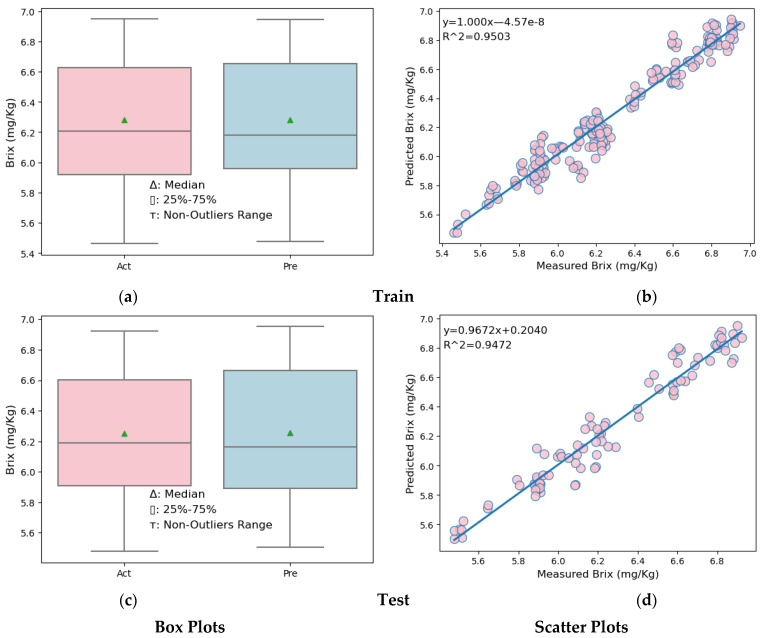
Box plots and scatter plots of the MLR model. (**a**) Box plot in the training partition of MLR (**b**) Scatter plot in the training partition of MLR. (**c**) Box plot in the testing partition of MLR. (**d**) Scatter plot in the testing partition of MLR.

**Figure 8:**
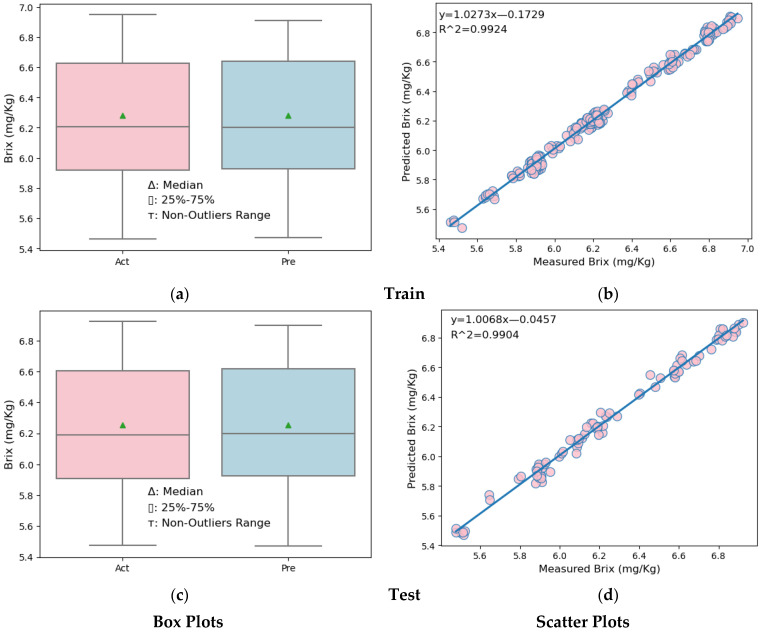
Box plots and scatter plots of the SVR model. (**a**) Box plot in the training partition of SVR (**b**) Scatter plot in the training partition of SVR. (**c**) Box plot in the testing partition of SVR. (**d**) Scatter plot in the testing partition of SVR.

Box plots and scatter plots for the training and testing sections of the MLR and SVR models are shown in Figure 7 and Figure 8.

Looking at the box plots (Figure 7a,c, left side) of both the training and test data partitions of the MLR model, it is seen that the upper quarter, lower quarter, and maximum of the predicted melon Brix values are slightly higher than the actual melon Brix values. In the scatter plots (Figure 7b,d, right side) showing the results of the melon Brix prediction models established for both the training and test data partitions, it is seen that the distribution model of the predicted and measured Brix values is close.

Similar comments can be made for the SVR model. Looking at the box plots (Figure 8a,c, left side) of both the training and test data partitions of the MLR model, it is seen that the upper quarter, lower quarter, and maximum of the predicted melon Brix values are slightly higher than the actual melon Brix values. However, when the SVR model and the MLR model are compared, the box plots produced with the SVR model results are closer and similar to the box plots produced with real values. In the scatter plots (Figure 8b,d, right side) showing the results of the melon Brix prediction models established for both the training and test data partitions, it is seen that the distribution model of the predicted and measured Brix values is very close. However, when the SVR model and the MLR model are compared, the observation values in the scatter plots showing the distributions of the actual and predicted Brix values of the SVR model are closer to the line. This shows us that the SVR model is better than the MLR model.

**Figure 9:**
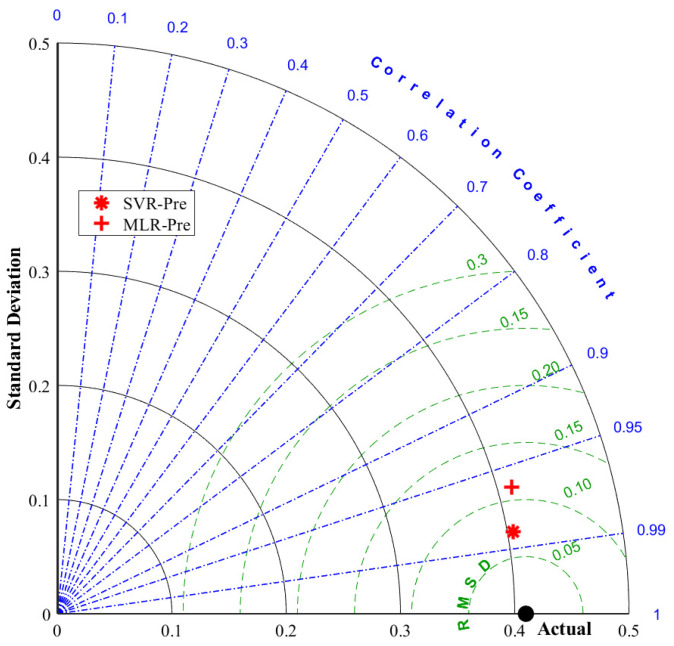
Taylor diagram of the SVR and MLR models for the test partition.

A Taylor diagram of the models is shown in Figure 9. The Taylor diagram is another criterion for comparing model performances. This graphical method uses the RMSD error criterion and the correlation coefficient, where the proximity to observed values identifies the best model. A model that is closer to the observed point is considered more accurate or better at representing the measured values [57,58]. As clearly seen in Figure 8, the Taylor diagram drawn for the test data set shows that the models that make the best predictions were obtained using the SVR method. 

In recent years, many researchers in the field of agriculture, as in many branches of science, have preferred machine learning techniques for solving various problems [46,59,60,61]. ML methods are more advantageous compared to the MLR method because they do not require statistical pretesting and do not require normal distribution properties of the data set [62]. Although there are many ML methods such as kNN, SVR, Random Forest, and Gradient Boosting, it is seen that in many studies, the ANN method is preferred for estimating the number of soluble solids, while the MLR method is preferred for comparing the results [46,63,64]. On the other hand, in some studies, the SVR method was preferred for estimating the amount of Brix [65,66]. In Fan et al.’s [65] study, the results of ML models are seen to be better than the partial least squares regression and Gaussian process regression model results. Of the two ML models used, the SVR model achieved better R^2^ and RMSE results than the Random Forest model. Similar results were obtained in another study comparing ML and regression models. The SVR model obtained a higher R^2^ value and lower RMSE value than the partial least squares regression model [66]. 

The results obtained from the conducted study can predict the amount of Brix in melon fruit with high accuracy using both ML and MLR methods. However, it is seen from the plots and metric results that ML methods are more successful than MLR. The important thing here is to choose the right ML method and make parameter settings (tuning) according to the type of problem.

## 4. Conclusions

In this article, the number of soluble solids in melon fruit was estimated by SVR and MLR methods and the results were compared. The regression equation was written according to the coefficients obtained as a result of the MLR analysis and the modeling results were evaluated with R^2^, MAE, MAPE, MSE, and RMSE metrics. According to the MLR model’s findings, while nitrogen, phosphorus, potassium, calcium, magnesium, zinc, manganese, copper, pH, dry matter amount, ash, phenol, sucrose, glucose, fructose, and fruit weight are significant, titratable acidity is insignificant. According to the testing data set, the MLR model results are MAE: 0.0728, MAPE: 0.0117, MSE: 0.0088, RMSE: 0.0936, and R^2^: 0.9472, and the ANN model results are MAE: 0.0334, MAPE: 0.0054, MSE: 0.0016, RMSE: 0.0398, and R^2^: 0.9904. Both models can be used to accurately predict the amount of Brix in melon fruit, but the SVR model predicts the amount of Brix in melon better than the MLR model. In addition, ANOVA testing showed no significant difference between the average Brix values according to melon rootstocks.

The findings argue that nutritional and physiological conditions such as macro-nutrients (nitrogen, phosphorus, potassium, calcium, and magnesium), micronutrients (zinc, manganese, and copper) and physical characteristics (pH, dry matter, and ash) significantly affect Brix values. Additionally, sucrose, glucose, and fructose were found to be highly and positively related to the soluble solids content. Other parameters such as fruit weight were also found to be significant predictors. Interestingly, titratable acidity was not significantly related to Brix values; it may not be a good predictor of soluble solids in melons. This finding helps streamline future testing protocols by potentially eliminating unnecessary measurements. The successful application of both MLR and SVR models, evaluated through multiple performance metrics (R², MAE, MAPE, MSE, and RMSE), provides growers and researchers with reliable tools for predicting melon quality. These models can be valuable for

- Optimizing cultivation practices;
- Improving rootstock selection;
- Enhancing quality control measures;
- Supporting decision-making in commercial melon production.

Further research could focus on validating these models across different melon varieties and growing conditions to establish their broader applicability in the field.

## Figures and Tables

**Table 1:** Required tests for the Multiple Linear Regression model, and the modeling results.

Diagnostic Tests	To Detect	Results/Interpretation
Multicollinearity	VIF	Since each VIF value < 10, there is no multicollinearity problem in the model.
Heteroscedasticity	Breusch–Pagan/Cook–Weisberg Test	Since *p* > 0.05, there is no heteroscedasticity in the model. Homoscedasticity refers to the variance of the error term being constant. The residuals are homoscedastic.
Linearity	Scatter plots and partial regression plots	The predictor variables exhibited a linear relationship with the predicted variable.
Normality	Shapiro–Wilk W Test	Since *p* > 0.05, both the residuals and the dependent variable show normal distribution.

**Table 2:** Information on the evaluation criteria.

Evaluation Criteria	Equation’s Depiction	Best/Worst Case
R^2^ Coefficient of Determination	$1-\frac{\sum_{i=1}^{m}{\left(Y_{i}-{\hat{Y}}_{i}\right)}^{2}}{\sum_{i=1}^{m}{\left(Y_{i}-\bar{Y}\right)}^{2}}$	1, 0
MAE Mean Absolute Error	$\frac{1}{m}\sum_{i=1}^{m}\left|Y_{i}-{\hat{Y}}_{i}\right|$	0, ∞
MAPE Mean Absolute Percentage Error	$\frac{1}{m}\sum_{i=1}^{m}\left|\frac{Y_{i}-{\hat{Y}}_{i}}{Y_{i}}\right|$	0, ∞
MSE Mean Square Error	$\frac{1}{m}\sum_{i=1}^{m}{\left(Y_{i}-{\hat{Y}}_{i}\right)}^{2}$	0, ∞
RMSE Root Mean Square Error	$\sqrt{MSE}$	0, ∞

**Table 3:** The results of the descriptive statistical analysis of the melon fruit.

Variables	Min	Max	SD	Mean	Variables	Min	Max	SD	Mean
Brix	5.46	6.95	0.40	6.27	pH	5.53	5.91	0.09	5.74
Nitrogen	1.17	3.62	0.58	2.17	Titratable Acidity	0.04	0.17	0.02	0.10
Phosphorus	0.13	0.46	0.06	0.26	Total Dry Matter	5.68	7.42	0.43	6.69
Potassium	1.74	2.76	0.25	2.20	Ash	0.31	0.49	0.04	0.40
Calcium	0.00	0.17	0.04	0.09	Total Phenolic	109.90	192.80	19.36	145.91
Magnesium	0.05	0.21	0.03	0.13	Sucrose	1.19	2.87	0.46	1.86
Zinc	2.46	9.86	2.01	5.29	Glucose	1.54	2.07	0.12	1.83
Manganese	1.39	5.45	1.05	2.50	Fructose	1.66	2.71	0.19	2.17
Copper	4.47	7.95	0.86	5.74	Fruit Weight	1.29	3.19	0.39	2.28

**Table 4:** Results of MLR.

Brix	Coefficient	Std. err.	*t*	*p* > |t|
Nitrogen (N)	−0.065	0.022	−2.930	0.004
Phosphor (P)	−0.690	0.223	−3.100	0.002
Potassium (K)	0.112	0.047	2.410	0.017
Calcium (Ca)	−2.419	0.340	−7.120	0.000
Magnesium (Mg)	1.970	0.391	5.040	0.000
Zinc (Zn)	−0.128	0.011	−11.760	0.000
Manganese (Mn)	0.207	0.021	9.950	0.000
Copper (Cu)	0.106	0.011	9.910	0.000
pH	0.655	0.156	4.200	0.000
Titratable Acidity	0.455	0.386	1.180	0.240
Dry Matter	0.212	0.043	4.930	0.000
Ash	−1.350	0.275	−4.910	0.000
T. Phenolic	0.003	0.001	5.000	0.000
Sucrose	0.617	0.048	12.730	0.000
Glucose	1.235	0.134	9.230	0.000
Fructose	0.089	0.046	1.920	0.056
Fruit Weight	−0.252	0.037	−6.850	0.000
Constant	−2.499	0.930	−2.690	0.008

**Table 5:** ANOVA test results.

Rootstocks	*n*	Mean	Std. Dev.	Min	Max	F	*p*
Sphinx	100	6.32	0.39	5.87	6.92	1.21	0.2997
Albatros	100	6.23	0.41	5.46	6.95		
Dinero	100	6.26	0.38	5.63	6.84		

**Table 6:** Metric results of MLR and ANN.

Method		MLR vs. SVR				
Evaluation Criteria		MAE	MAPE	MSE	RMSE	R^2^
Partition	Training	0.0675/0.0299	0.0108/0.0048	0.0076/0.0012	0.0871/0.034	0.9503/0.9924
	Testing	0.0728/0.0334	0.0117/0.0054	0.0088/0.0016	0.0936/0.0398	0.9472/0.9904

## Data Availability

The raw data supporting the conclusions of this article will be made available by the authors on request.
